# A Gravesend Good Samaritan

**Published:** 1915-02-27

**Authors:** 


### A Gravesend Good Samaritan.

PRESENTATION TO MRS. WHITCOMBE.

The members of the Ladies' Association met on Friday last week in the Board Room at the Gravesend Hospital to do honour to Mrs. Whitcombe, who for the past quarter of a century has acted as hon. secretary and treasurer to the Gravesend Hospital Samaritan Society, and who has relinquished the work on leaving Gravesend to take up her residence in London. Mrs. Whitcombe's long and energetic work for the Society is well known, and she will be sorely missed by countless numbers of the sick poor, not only in the borough, but around the whole country-side. It was to Mrs. Whitcombe that the poor flocked when in need of temporary relief, and when aid was wanted by families whose natural supporters lay, through accident or sickness, in the hospital. Through the Society, Mrs. Whitcombe was able to provide clothing for destitute patients who from long illness had been reduced to poverty, and whose circumstances justified such assistance and nourishment; while hundreds of patients have benefited by being sent away to the sea for convalescence.

It was perhaps in this particular branch of the work that Mrs. Whitcombe was best known to the poor, for every year between seventy and eighty debilitated patients, whose restoration to health was impracticable in the hospital or at their own ill-provided and often unhealthy homes, were sent away to various convalescent homes, where the pure air, rest, and nutrition restored the patients once again to health. Mrs. Whitcombe did not mind where the patient hailed from; in fact, quite half the patients came from Northfleet and Perry Street and the surrounding villages on both sides of the river. No deserving case was every barred from her assistance, for she helped the poor by providing money for nourishment, coal, surgical appliances, water-beds and air-pillows, elastic stockings and bandages, warm clothing, and the like.

Mrs. Whitcombe was particularly interested in the maternity side of the work, and many mothers have been thankful to her for boxes of clothes lent and for kind help at a time of great need. A glance at the twenty-ninth annual report of the Samaritan Society will speak for itself of the admirable work done—a work which proves a valuable adjunct to the work of the hospital. It shows that considerably over £150 was expended during 1914 on the alleviation of distress and in helping the sick during that year. When this is compared with the £8 expended during the first year Mrs. Whitcombe took over the work—in 1889—it will be ample evidence to show the growth of the great work in which she has been so actively and wholeheartedly engaged.

Her work is to be carried on, for the present, by Mr. W. Pearson, the hospital secretary, and it is Mrs. Whitcombe's earnest wish that he should receive the same support as kind friends have been wont to give to her.

The Ladies' Association had before their meeting a handsomely bound volume of photographs of the various departments of the hospital; on the centre of the cover of which was Mrs. Whitcombe's initials and at opposite corners the years 1889-1914, the year of Mrs. Whitcombe taking over the work and the year of her relinquishing the same. The volume, which was presented to Mrs. Whitcombe in London by the Chairwoman, Mrs. Snelling, contains a complimentary preface, followed by the signatures of the ladies present and of the management committee of the hospital—about one hundred in all.

